# Free-view gait recognition

**DOI:** 10.1371/journal.pone.0214389

**Published:** 2019-04-16

**Authors:** Yonghong Tian, Lan Wei, Shijian Lu, Tiejun Huang

**Affiliations:** 1 National Engineering Laboratory for Video Technology, School of Electronics Engineering and Computer Sciences, Peking University, Haidian, China; 2 School of Computer Science and Engineering, Nanyang Technological University, Singapore, Singapore; 3 Pengcheng Laboratory, Shenzheng, China; University of Zaragoza, SPAIN

## Abstract

Human gait has been shown to be an effective biometric measure for person identification at a distance. On the other hand, changes in the view angle pose a major challenge for gait recognition as human gait silhouettes are usually different from different view angles. Traditionally, such a multi-view gait recognition problem can be tackled by View Transformation Model (VTM) which transforms gait features from multiple gallery views to the probe view so as to evaluate the gait similarity. In the real-world environment, however, gait sequences may be captured from an uncontrolled scene and the view angle is often unknown, dynamically changing, or does not belong to any predefined views (thus VTM becomes inapplicable). To address this *free-view gait recognition* problem, we propose an innovative view-adaptive mapping (VAM) approach. The VAM employs a novel walking trajectory fitting (WTF) to estimate the view angles of a gait sequence, and a joint gait manifold (JGM) to find the optimal manifold between the probe data and relevant gallery data for gait similarity evaluation. Additionally, a RankSVM-based algorithm is developed to supplement the gallery data for subjects whose gallery features are only available in predefined views. Extensive experiments on both indoor and outdoor datasets demonstrate that the VAM outperforms several reference methods remarkably in free-view gait recognition.

## Introduction

In recent years, surveillance cameras have been widely deployed in many cities. To automatically analyze the data captured from these cameras (e.g., for searching for a suspicious person or vehicle), different biometric technologies have been developed and playing more and more important roles in public security applications and crime investigation. Human gait is one of the well-recognized biometric features to ascertain the identity of a human at a distance [1, 2]. On the other hand, human gait may be affected by various factors in practical visual surveillance scenes, e.g. change in view angles, variation of walking speed, carrying an object and even wearing different types of shoes [3]. Among all these factors, change in view angles is regarded as one of the most common challenges as it often changes the visual features significantly (e.g., visible body parts, global shape statistics, and walking trajectories [4, 5]).

**Fig 1:**
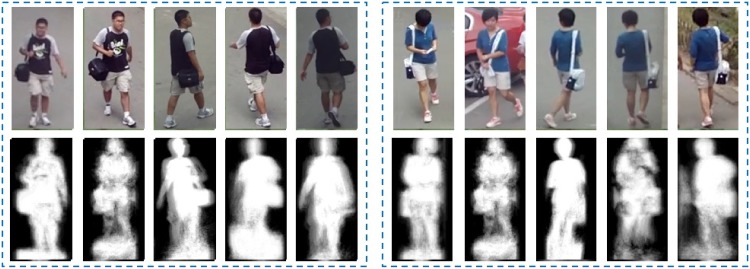
Within free-view scenes with weak control and definition, both probe and gallery gait sequences could be captured from arbitrary views. For example, as shown in this figure, the two groups of samples from the PKU HumanID Gait Dataset [12] has low-quality gait features.

Though individual gaits often vary across views, they are still correlated and share certain view-invariant gait features [6, 7]. The gait recognition problem has been investigated under three typical setups [4]: 1) *fixed-view* gait recognition where both probe and gallery gaits are captured from the same view; 2) *cross-view* gait recognition where the probe and gallery gaits are captured from different views; and 3) *multi-view* gait recognition where the probe gaits under a specific view are recognized by gallery gaits from multiple views. All three setups assume that gaits are well defined and captured within a well-controlled environment with few background clutters. On the other hand, the probe gaits may be captured from *uncontrolled* scenes under *arbitrary* views in practical situations, and some of them even do not have any matched views in the gallery dataset. Moreover, the view angles in a probe gait sequence may be dynamically changing since the pedestrian may walk freely as illustrated in Fig 1. Most existing gait recognition techniques do not work well under such arbitrary free view scenario due to the very different setups.

The *free-view* gait has two unique features as illustrated in Fig 1. First, it is captured under arbitrary view angles with large human pose variations that directly lead to low gait regularity (i.e. the regular relationship between gait features of a pair of views with certain transformation between them). Second, it is often associated with cluttered background and sometimes occlusions that directly lead to silhouette noises and low gait feature quality. We develop an innovative view-adaptive mapping (VAM) that tackles the free-view gait recognition challenge from three aspects. The first is automatic recognition of the view angle of a gait sequence. For this we design a novel walking trajectory fitting (WTF) that estimates gait views by first analyzing the walking trajectory of a pedestrian and then calculating the corresponding view angle. The second is automatic approximation for undefined probe views. For this we design a joint gait manifold (JGM) that finds the optimal manifold between the probe data and relevant gallery data for gait similarity evaluation.

The third is gallery gait data of incomplete views, which is common for most gait datasets due to the difficulty in collecting gallery data of all subjects from all predefined views. Similar to other gait recognition methods such as View Transformation Model (VTM) [2, 8, 9] and Canonical Correlation Analysis (CCA) [10], VAM also favors complete gallery data under all predefined views. It addresses this problem by a novel RankSVM-based algorithm that supplements the gallery data for subjects whose gallery features are only partially available under certain predefined views. Specifically, it supplements the gallery gait features of a subject by using the gait features of neighboring subjects under the same view, as well as the same subject’s gait features of the closest views. It thus formulates the subject neighborhood measure as a learn-to-rank problem and exploits RankSVM to learn the optimal ranking function.

The proposed VAM was evaluated over two gait datasets. The first is CASIA gait dataset B [11] that was created for benchmarking multi-view gait recognition in controlled indoor environments. We construct two variants of this dataset for evaluation within controlled free-view scenes. The second is the PKU HumanID gait dataset [12] that is a free-view dataset captured in an uncontrolled outdoor environment.

## Related work

This section reviews the related work briefly. Existing gait recognition techniques can be broadly grouped into three categories, to be discussed in the following three subsections.

### Gait recognition based on view synthesis

View synthesis based approach aims to generate virtual views for optimal gait recognition. For example, [13] presents a view normalization method for multi-view face and gait recognition, where a set of monocular views are utilized to construct image-based visual hull (IBVH) and render virtual views for gait recognition. [14] exploits hard and soft kinematic constraints for 3D tracking and gait pattern extraction from human motion data. [15] sets up a human 3D model from video sequences captured by multiple cameras for gait tracking and recognition. [16] uses an active vision sensor to capture 3D data and then synthesizes the complete gait sequence by interpolation of joint positions and their movements from the fitted body models. [17] describes a silhouette-based method that uses viewpoint projection to convert 3D data into 2D view-invariant data. [18] employs articulated cylinders with 3 Degrees of Freedom (DoF) at each joint to model the human lower legs and then extracts structural and dynamic 3D gait features.

In addition, [19] presents a multi-view gait recognition method that exploits 3D morphological gait sequence analysis to extract gait descriptors and then classifies their temporal patterns using Support Vector Machine (SVM). [20] proposes the arbitrary view transformation to address the problem where a probe view is excluded from views for the training subjects, by reconstructing the 3D models for the training subjects. [21] solves the walking direction change problem in gait recognition by estimating the walking direction for each subject and synthesizing a virtual image corresponding to the estimated direction from a 4D gait database. [22] instead synthesizes an image generated from 3D volumes after estimating the local walking direction in the first and second parts of a gait cycle.

The view synthesis based gait recognition is often complex and not easy to implement because it requires a fully controlled and cooperative multi-camera system to either reconstruct the 3D gait model or synthesize virtual view images directly.

### Gait recognition based on view-invariant features

Quite a number of gait recognition systems have been reported to make use of view-invariant features. For example, [23] presents a perspective projection model that generates a side view from any arbitrary view using a single camera. [24] and [25]) integrate information from multiple views to extract view-invariant features. [26] proposes a three-layer scheme using bilinear models, where image sequences are mapped to observation vectors using Markov modeling. [27] introduces a gait recognition approach by computing view-normalized trajectories of body parts from monocular video sequences. [28] introduces a local binary pattern (LBP) flow as a static representation of gait movement, and shows very promising results in gait recognition. [6] utilizes angular measurements and trunks spatial displacement as a view-invariant gait feature for view-independent gait biometrics. [7] introduces a normal distance map as a robust gait feature descriptor by combining the distance transform with curvatures of local contours. [29] instead normalizes gaits from arbitrary views by utilizing the invariant low-rank textures (TILTs) for view-invariant gait feature extraction. Some auxiliary data from other sensors such as accelerometer [1] and RGBD [30] have also been utilized for robust gait recognition.

Inspired by the great successes of deep convolutional neural networks (CNNs) in image recognition tasks, several methods (e.g., [31–34]) have been proposed in recent year which utilize CNNs to learn more robust gait representations. For example, [31] tackles multi-view gait recognition by training a 3D CNN by using grayscale images and optical flow as input. [32] trains CNN and Siamese neural networks [33] by using gait energy images. [34] proposes a similarity learning approach for gait-based human identification via CNNs. The CNN-based gait recognition achieve reliable performance even under large view differences as long as a large number of training examples are available, demonstrating great potentials for future study and applications.

### Gait recognition based on view transformation

Different with the techniques using view synthesis or view-invariant features, the view transformation based techniques recognizes gaits by learning the mapping/projection relationship of gait features across views. For example, [8] introduces View Transformation Model (VTM) to match gait features of different walking directions. [9] improves the VTM by using gait energy image (GEI) features and Linear Discriminant Analysis (LDA) for GEI feature optimization. In addition, [2] improves VTM from a different approach by incorporating a score normalization framework with quality measures that evaluate how well the gait features of test subjects are represented by a joint subspace spanned by a set of gait features of training subjects.

To overcome the constraint of the VTM that often requires a large training dataset, [35] re-formulates the VTM construction as a regression problem, and applies Support Vector Regression (SVR) to create the VTM. [36] instead learns the LDA-subspaces to extract discriminative information from gait features. Similarly, [37] introduces a robust VTM via Principal Component Analysis (PCA), where gait features are extracted by adopting the feature selection method with Partial Least Square (PLS) on the original GEI. [10] models the correlation of gait sequences from different views using Canonical Correlation Analysis (CCA), where gait sequences from two views are projected into two different subspaces such that they could be maximally correlated. The view transformation based techniques have demonstrated superior gait recognition performance, but they require the gait data across all predefined views to train the VTM or CCA which makes them impractical in many real-world applications.

## The proposed method

We define the *free-view gait recognition* as a special gait recognition problem, where gait sequences are captured within an uncontrolled scene and the probe view angles could be unknown, dynamically changing, or without any match within the predefined gallery views. This gait recognition problem can be boiled down to three sub-problems, i.e., how to automatically estimate the view angles, how to deal with undefined probe view angles over which gait similarity can be evaluated properly, and how to supplement gallery gait features when they are incomplete with respect to predefined views.

**Fig 2:**
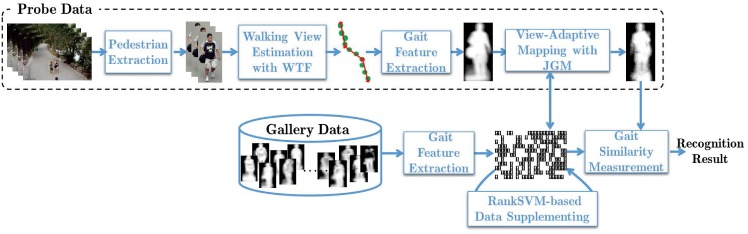
The framework of our proposed view-adaptive mapping (VAM) technique for free-view gait recognition.

The overview framework of our approach in Fig 2 shows how we address the three sub-problems. For the first sub-problem, we perform gait period analysis and gait view estimation to extract more robust gait features in the real-world scenes. In particular, we propose a novel walking trajectory fitting (WTF) algorithm to estimate the walking view. The basic idea is to fit the walking trajectory in one gait circle as a straight line, and use the line’s angle against the camera’s direction to identify the view angle in that circle, more details to be described in Gait View Estimation. For the second sub-problem, we treat the observations of human gaits from adjacent views as multiple manifolds that share the same parameter space, and introduce the *joint gait manifold* (JGM) to model the dependencies present in a variety of gait features across views. The JGM generates an optimal joint manifold for gait features from two gallery views that are closest to the probe view angle, on which the probe and relevant gallery gait data can be directly compared, more details to be described in Joint Gait Manifold. For the third sub-problem, a RankSVM-based algorithm is introduced to supplement the gallery gait features to cope with the large difference between the probe view and gallery data, more details to be described in RankSVM-based Gallery Data Supplementing.

### Gait view estimation

In the gait view estimation, pedestrians are first located in the captured videos. Gait periods are then extracted by analyzing the normalized auto correlation (NAC) of gait silhouettes [8]. WTF is finally applied to estimate the pedestrian’s walking angle.

#### Pedestrian extraction

Given a gait sequence in a video, the foreground pedestrian can be extracted from every frame by using a Gaussian mixture model (GMM) based foreground extraction algorithm [38]. This GMM based algorithm is robust to changes of the observed scene and simple to implement, but often introduces noisy foreground pixels due to abrupt lighting changes. In our system, morphological operations [39] and foreground connected component analysis [40] are introduced for noise elimination. The Faster-RCNN-based pedestrian detector [41] is applied to detect pedestrians, where a particle filter based object tracker with an optimized observation model is used to obtain the pedestrian’s walking trajectory [42].

#### Gait period analysis

**Fig 3:**
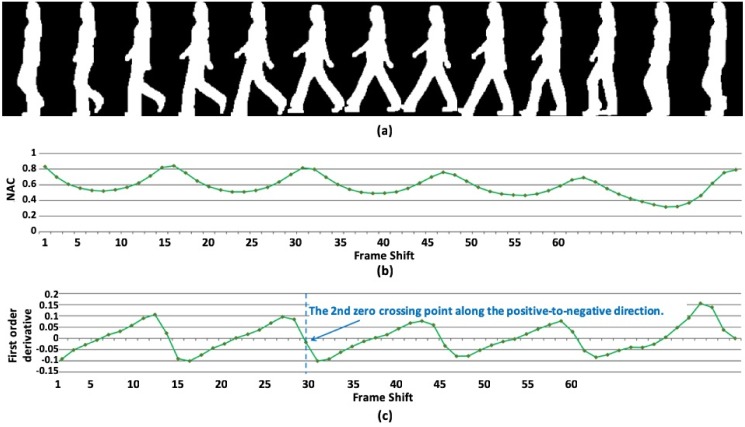
Gait period analysis: (a) The gait silhouettes for the extracted figure-centric images of a walking person; (b) The estimated NACs; and (c) The first-order derivative curve of NACs.

Human gait can be generally treated as a periodic motion and expressed using the gait cycle or stride. A complete gait period consists of two steps, where each step denotes the motion between successive heel strikes of opposite feet [43]. Given a sequence of the extracted silhouettes, gait period can thus be detected by maximizing the NAC of the size-normalized silhouette images along the temporal axis. Let *s*^*m*^(*x*, *y*, *i*) be the pixel value of the *m*^*th*^ person’s gait silhouette at position (*x*, *y*) of the *i*^*th*^ frame, the autocorrelation factor *C*^*m*^(*t*) under *t* frame shift can be calculated by [8]

$$\begin{array}{c} C^{m}\left(t\right)=\frac{\sum_{x,y}\sum_{i=0}^{N_{t}}s^{m}\left(x,y,i\right)s^{m}\left(x,y,i+t\right)}{\sqrt{\sum_{x,y}\sum_{i=0}^{N_{t}}s^{m}{\left(x,y,i\right)}^{2}}\sqrt{\sum_{x,y}\sum_{i=0}^{N_{t}}s^{m}{\left(x,y,i+t\right)}^{2}}}, \end{array}$$ (1)

where *N*_*t*_ = *N*_*total*_ − *t* − 1, *N*_*total*_ is the total number of frames in the sequence. Since a gait period consists of two steps, it can be naturally estimated as frame shift corresponding to the second peak of the NAC. Fig 3 shows an example of gait period analysis using NACs, where the period transition position is defined at the zero-crossing point along the positive-to-negative direction. The gait period *T*_*gait*_ can thus be determined by the 2^nd^ period transition position.

**Fig 4:**
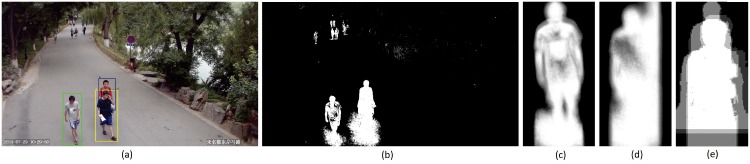
An example of gait silhouettes extracted from a real free-view scene: (a) A sample frame from the Camera WMHD in the PKU HumanID dataset, where three pedestrians are labeled using color bounding boxes (green for subject 0, yellow for subject 6 and blue for subject 8); (b), (c) and (d) The extracted gait silhouettes for the three subjects.

We estimate gait periods by analyzing the whole gait silhouette due to its better robustness as compared with aspect ratio [9, 15, 43]. Fig 4 shows an example, where shadow and occlusion exist in the extracted silhouettes. In this case, it is difficult to estimate the gait period precisely by using the aspect ratio of the silhouette bounding boxes which has little change across several consecutive frames. Comparatively, the whole gait silhouettes still demonstrate clear change due to the continuous motion.

#### Gait view estimation

Instead of estimating views on the extracted gait features [10], we first conduct trajectory analysis to estimate the pedestrian’s walking view before feature extraction. Specifically, the barycenter of a person is tracked as his/her walking trajectory, and the trajectory within several adjacent gait cycles is approximated as a straight line. The gait view can thus be estimated by calculating the angle between his/her working lines and the camera’s observation direction. Note that the observation view direction for a given camera can be obtained from the camera’s calibration information by using some existing calibration method, e.g., [44].

Firstly, the barycenter of the *m*^*th*^ person at the *t*^*th*^ frame, expressed as a 2-D column vector, is calculated as the weighted center of the gait silhouette:

$$\begin{array}{c} b^{m}\left(t\right)=(\begin{array}{c} \frac{1}{\sum_{x=0}^{W}\sum_{y=0}^{H}s^{m}(x,y,t)}\sum_{x=0}^{W}\sum_{y=0}^{H}x\times s^{m}(x,y,t)\\ \frac{1}{\sum_{x=0}^{W}\sum_{y=0}^{H}s^{m}(x,y,t)}\sum_{x=0}^{W}\sum_{y=0}^{H}y\times s^{m}(x,y,t) \end{array}), \end{array}$$ (2)

where *W* and *H* are the width and height of the bounding box of the current silhouette. Here *s*^*m*^(*x*, *y*, *t*) is 255 for foreground person pixels 0 for background pixels.

The walking trajectory can be generated within a gait period once the barycenter is available. Specifically, the straight line of the *i*^*th*^ gait cycle can be estimated by:

$$\begin{array}{c} \underset{k_{i,1},k_{i,2},k_{i,3}}{\arg\min}\sum_{t=0}^{T_{gait}}{\left[\begin{array}{c} k_{i,1}\\ k_{i,2} \end{array}\right]}^{T}b^{m}\left(i\times T_{gait}+t\right)+k_{i,3} \end{array}$$ (3)

where {*k*_*i*,*j*_|*j* = 1, …, 3} are three parameters, T denotes matrix transpose and *T*_*gait*_ is the frame number of each estimated gait period. As *k*_*i*,3_ does not affect the observed view angle between the walking trajectory and the direction of the camera, parameters {*k*_*i*,*j*_|*j* = 1, 2} are used to represent the estimated *i*^*th*^ walking trajectory line.

**Algorithm 1**: The walking trajectory fitting (WTF) algorithm.

**Input**: the pre-defined threshold *Th*_*θ*_,

   the total number of gait periods in the sequence *T*_*total*_,

   the frame number of each gait period *T*_*gait*_;

**Output**: the number of the estimated walking trajectory lines *L*, each with parameters {*k*_*i*,*j*_|*j* = 1, 2}_*i*∈[1 …*L*]_.

*i* = 0;

**while** *i* < *T*_*total*_ **do**

 *w* = 0;

 **while** *k*_*i*,1_ = *k*_*i*+*w*+1,1_ = 0 or $|\frac{k_{i,2}}{k_{i,1}}-\frac{k_{i+w+1,2}}{k_{i+w+1,1}}|<Th_{\theta }$ **do**

  *w* = *w* + 1;

 **end**

 **if** *w* > 0 **then**

  $\underset{k_{i,1},k_{i,2}}{\arg\min}\sum_{t=0}^{\left(w+1\right)\times T_{gait}}{\left[\begin{array}{c} k_{i,1}\\ k_{i,2} \end{array}\right]}^{T}b^{m}\left(i\times T_{gait}+t\right)$;

  *i* = *i* + *w*;

 **end**

 *i* = *i* + 1;

**end**

*L* = *i*;

After segmenting a walking sequence into end-to-end lines, a smoothing strategy is applied to merge adjacent lines. As described in Algorithm 1, adjacent gait cycles are regarded to be under the same view angle if they meet the condition *k*_*i*,1_ = *k*_*i*+*w*+1,1_ = 0 or $|\frac{k_{i,2}}{k_{i,1}}-\frac{k_{i+w+1,2}}{k_{i+w+1,1}}|<Th_{\theta }$, where *Th*_*θ*_ is the pre-defined threshold and *w* + 1 is the size of the current smoothing window. Adjacent gait cycles will thus be re-fitted into a single line if the angle difference between their walking lines is less than *Th*_*θ*_. This procedure repeats until there are no more adjacent walking lines to be merged. In our implemented system, *Th*_*θ*_ is empirically set to 15°.

**Fig 5:**
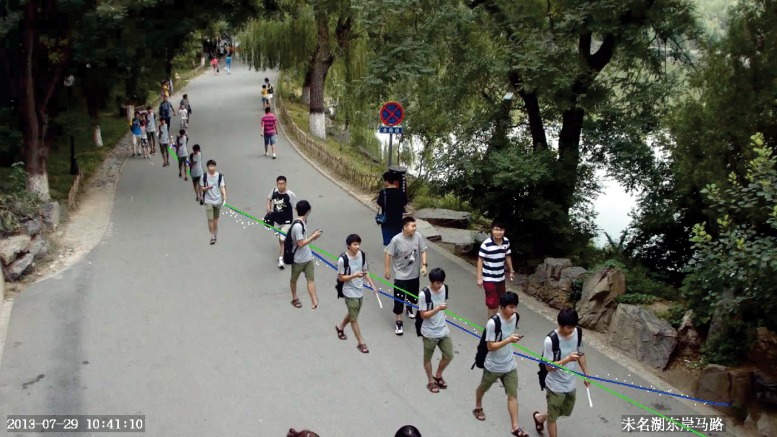
Illustration of the WTF algorithm by using the man wearing a grey T-shirt: The blue line represents his walking trajectory in several gait cycles, while the green one denotes the fitted walking line in the gait period. For better visualization, all his silhouette images in a gait period are manually superposed in one picture.

Note the position of successive barycenters may have little change. One possible reason is that the person is walking under the frontal or back view of the camera. Under such circumstance, the silhouette size change can tell the walking status, e.g., the silhouette size will become larger when the person is walking towards the camera under the frontal view. Fig 5 visualizes the WTF based gait view estimation, where the man wearing a grey T-shirt with a backpack is the target person, the blue line represents his original walking trajectory and the green line denotes the fitted walking line in the gait period. Under such configuration, the view angle between the green line and the camera direction can be used to estimate the walking view.

Compared with view classification based on Gaussian Process (GP) [10] or SVM, our WTF based method can effectively handle challenging situations when a person walks freely, e.g. when he/she changes the walking direction. In addition, both GP-based and SVM-based methods leverage the analysis of Truncated GEIs which are prone to shadows in silhouette images as shown in Fig 4. Our WTF based method is instead more robust under complex scenes in real-world surveillance videos.

### Joint gait manifold

In the joint gait manifold (JGM), human gaits from several adjacent views are treated as multiple manifolds that share the same parameter space. The JGM models the dependency of gait features across views and generates optimal joint manifolds for gait features of closest gallery views to the probe view for proper comparison.

#### Gait feature extraction

**Fig 6:**
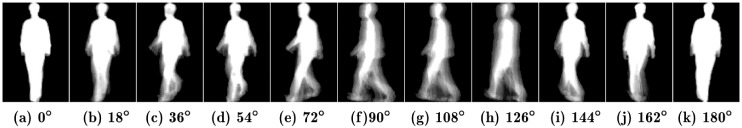
Examples of the GEI features under different views in the CASIA dataset B.

We use the GEI feature as the original gait feature due to its robustness to silhouette errors and image noises [45]. GEI at position (*x*, *y*) is defined as:

$$\begin{array}{c} g(x,y)=\frac{1}{T_{gait}}\sum_{q=0}^{Q}\sum_{t=0}^{T_{frame}}s(x,y,t+q\times T_{gait}), \end{array}$$ (4)

where *s*(*x*, *y*, *t*) is a pixel located at (*x*, *y*) of the *t*^*th*^ (*t* = 1, 2…*T*_*frame*_) silhouette image in the *q*^*th*^ (*q* = 1, 2…*Q*) gait cycle. Here all *Q* gait cycles should be under the same view angle. All silhouettes are re-scaled to a fixed width (denoted by *W*) and height (denoted by *H*). The original GEI feature thus becomes an *W* × *H*-dim vector. Linear Discriminant Analysis (LDA) is then applied to obtain an *N*_*g*_-dim vector. Fig 6 shows examples of this gait feature for a person under different views.

#### Gait similarity evaluation

The gait similarity between the probe and relevant gallery gait features under the same view can be evaluated after the gait feature extraction. In VTM [46], a view transformation matrix is constructed from the training data using Singular Value Decomposition (SVD). Let *N* and *M* denote the numbers of the pre-defined views and the subjects, $g_{n}^{m}$ denote the *N*_*g*_-dim gait feature of subject *m* under the *n*^*th*^ view angle *θ*_*n*_, and **v**^*m*^ denote the intrinsic gait feature of subject *m* for any view angle (i.e., view-invariant feature). The view transformation matrix can be denoted as **P** = [**P**_1_, …, **P**_*N*_]^T^ [9], where **P**_*n*_ is the *N*_*g*_ × *M* subject-independent matrix that projects the intrinsic feature vector **v**^*m*^ to the gait feature vector $g_{n}^{m}$ under the view angle *θ*_*n*_ as follows:

$$\begin{array}{c} g_{n}^{m}=P_{n}v^{m}. \end{array}$$ (5)

So gait feature transformation from view angle *θ*_*j*_ to view angle *θ*_*i*_ can be derived by:

$$\begin{array}{c} {\hat{g}}_{j|i}^{m}=P_{i}P_{j}^{+}g_{j}^{m}, \end{array}$$ (6)

where $P_{j}^{+}$ is pseudo inverse of **P**_*j*_, and ${\hat{g}}_{j|i}^{m}$ is the transformed feature of $g_{j}^{m}$ on *θ*_*i*_.

To address the problem that the probe view angle *θ*_*i*_ does not belong to any predefined gallery views, we treat the observations of human gaits of adjacent views as multiple manifolds that share the same parameter space, and introduce the *joint gait manifold* (JGM) to model the dependency present in gait features across views. Let $\theta_{j_{1}}$ and $\theta_{j_{2}}$ denote the two gallery view angles closest to *θ*_*i*_ (let $\theta_{j_{1}}\leq \theta_{i}\leq \theta_{j_{2}}$ without loss of generality), $M_{j_{1}}$ and $M_{j_{2}}$ be their corresponding gait manifolds. Suppose the probe gait feature **g**_*i*_ is collected from an unknown manifold $N_{i}$, our objective is to learn an optimal joint manifold $M^{*}\subset M$ where $M=M_{j_{1}}\times M_{j_{2}}$ denotes the product manifold [47] that satisfies the following two conditions: 1) the local geometries inside $M_{j_{1}}$ and $M_{j_{2}}$ will be preserved in $M^{*}$, and 2) the distance between $N_{i}$ and $M^{*}$ will be less than the distance between $N_{i}$ and any other manifold in M. The first condition ensures that $M^{*}$ is locally homeomorphic to $M_{j_{1}}$ and $M_{j_{2}}$. The second ensures that $M^{*}$ is the optimal joint manifold and can be used to approximate the unknown manifold $N_{i}$ (denoted by $M^{*}\approx N_{i}$).

**Fig 7:**
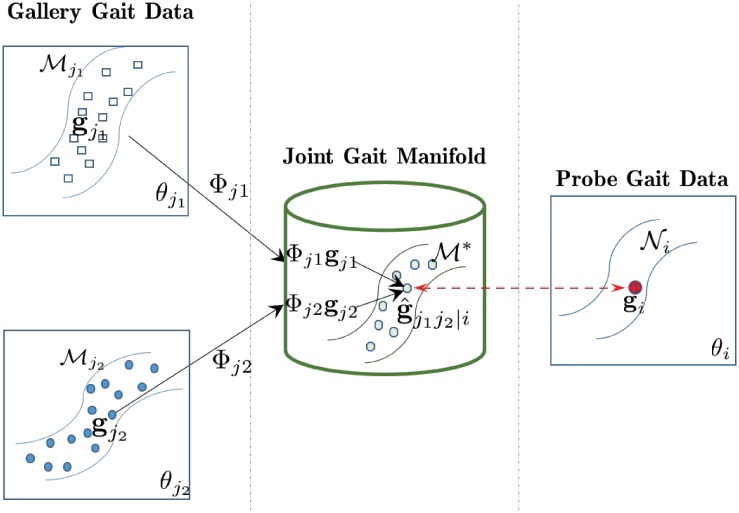
Illustration of joint gait manifold.

To obtain a joint manifold that satisfies the first condition, [47] suggests to project each component manifold into a lower-dimensional subspace through random projection. Under our setting, it can be expressed by

$$\begin{array}{c} {\hat{g}}_{j_{1}j_{2}|i}=\Phi^{*}{\left[g_{j1}g_{j2}\right]}^{T}=\left[\Phi_{j_{1}}\Phi_{j_{2}}\right]{\left[g_{j_{1}}g_{j_{2}}\right]}^{T}=\Phi_{j_{1}}g_{j_{1}}+\Phi_{j_{2}}g_{j_{2}}, \end{array}$$ (7)

where $\Phi^{*}=\left[\Phi_{j_{1}}\;\Phi_{j_{2}}\right]$, and $\Phi_{j_{1}}$ (or $\Phi_{j_{2}}$) is the projection matrix in $\theta_{j_{1}}$ (or $\theta_{j_{2}}$). Fig 7 illustrates this idea, where the transformed gallery gait vector ${\hat{g}}_{j_{1}j_{2}|i}$ on the joint manifold can be directly compared with **g**_*i*_. Note that this idea can be easily extended to the case of *k* component manifolds.

One key problem here is how to chose the projection matrix Φ*. Instead of using random projection in [47], we exploits the manifold alignment algorithm in [48] which learns two linear mapping matrices that can project two manifolds to one space so that instances (from different manifolds) with similar local geometry will be mapped to similar locations. Technologically, this algorithm formulates the manifold alignment problem as a generalized eigenvalue decomposition (GEVD) problem. It can be easily shown that $\Phi_{j_{1}}=P_{i}P_{j_{1}}^{+}$ and $\Phi_{j_{2}}=P_{i}P_{j_{2}}^{+}$ are exactly the mapping matrices satisfying this problem, where **P**_*i*_ (or **P**_*j*_) is the subject-independent matrix extracted from the view transformation matrix [9] that is constructed from the training dataset with several available viewing angles, and $P_{j}^{+}$ is the pseudo inverse matrix of **P**_*j*_.

Once $\Phi_{j_{1}}$ and $\Phi_{j_{2}}$ are available, the remaining problem is whether the joint manifold $M^{*}=\left\{\Phi_{j_{1}}g_{j_{1}}+\Phi_{j_{2}}g_{j_{2}}:g_{j_{1}}\in M_{j_{1}},g_{j_{2}}\in M_{j_{2}}\right\}$ is the optimal one that can approximate $N_{i}$. To evaluate this, a direct idea is to check whether the distance between $N_{i}$ and $M^{*}$ is less than that between $N_{i}$ and any other manifold in M. Since the probe gait manifold $N_{i}$ is unknown in the free-view setting, we use the available probe data that are captured from the same view angle *θ*_*i*_ to simulate $N_{i}$. Let $M^{*}=\left\{\alpha_{j_{1}j_{2}|i}\Phi_{j_{1}}g_{j_{1}}+\left(1-\alpha_{j_{1}j_{2}|i}\right)\Phi_{j_{2}}g_{j_{2}}:g_{j_{1}}\in M_{j_{1}},g_{j_{2}}\in M_{j_{2}}\right\}$ denote the general form of the joint mainifold of $M_{j_{1}}$ and $M_{j_{2}}$ where *α*_*j*_1_*j*_2_|*i*_ is a weighting factor with 0 ≤ *α*_*j*_1_*j*_2_|*i*_ ≤ 1. As in [47], three distance measures can be used including *minimum separation distance*, *maximum separation distance* and *Hausdorff distance*. Among them, the minimum separation distance establishes the lower bound that guarantees the maximal similarity between the component manifolds. We therefore adopt it to measure the distance between two gait manifolds as follows:

$$\begin{array}{c} \begin{array}{cc} \delta (M^{*},N_{i}) & =\underset{{\hat{g}}_{j_{1}j_{2}|i}^{*}\in M^{*},g_{i}\in N_{i}}{\inf}d({\hat{g}}_{j_{1}j_{2}|i}^{*},\;g_{i})\\ & =\underset{g_{j_{1}}\in M_{j_{1}},g_{j_{2}}\in M_{j_{2}},g_{i}\in N_{i}}{\inf}d\left(\alpha_{j_{1}j_{2}|i}\Phi_{j_{1}}g_{j_{1}}+\left(1-\alpha_{j_{1}j_{2}|i}\right)\Phi_{j_{2}}g_{j_{2}},\;g_{i}\right) \end{array}\;\;\;, \end{array}$$ (8)

where inf represents the infimum and *d*(⋅, ⋅) denotes the distance between two gait features in the same manifold. Here we use the simple L1-norm distance:

$$\begin{array}{c} d\left(g_{i},g_{j}\right)=∥g_{i}-g_{j}∥. \end{array}$$ (9)

A smaller value *d*(**g**_*i*_, **g**_*j*_) means larger similarity between **g**_*i*_ and **g**_*j*_. As a result, the optimal joint manifold can be obtained by:

$$\begin{array}{c} \begin{array}{cc} \alpha_{j_{1}j_{2}|i}^{*} & =\arg\min\;\;\delta (M^{*},N_{i})\\ & =\underset{0\leq \alpha_{j_{1}j_{2}|i}\leq 1}{\arg\min}\underset{\overset{\overset{g_{j_{1}}\in M_{j_{1}},}{g_{j_{2}}\in M_{j_{2}},}}{g_{i}\in N_{i}}}{\inf}d\left(\alpha_{j_{1}j_{2}|i}\Phi_{j_{1}}g_{j_{1}}+\left(1-\alpha_{j_{1}j_{2}|i}\right)\Phi_{j_{2}}g_{j_{2}},\;g_{i}\right) \end{array}. \end{array}$$ (10)

For simplicity, we initialize *α*_*j*_1_*j*_2_|*i*_ at 0.5 and perform bi-directional linear search with the step length of 0.05 to find its optimal value.

The JGM algorithm can be summarized in Algorithm 2. Given the training dataset with several available viewing angles, the view transformation matrix can be constructed offline [9]. Then given one probe gait sequence, the corresponding view angle is first estimated, and two mapping matrices $\Phi_{j_{1}}$ and $\Phi_{j_{2}}$ can be calculated. After that, the optimal joint manifold $M^{*}$ is derived by learning $\alpha_{j_{1}j_{2}|i}^{*}$ through Eq 10, on which the gait similarity can be directly evaluated. Note that in the free-view setting, a probe gait sequence typically consists of several gait features extracted from different view angles (one example is shown in Fig 5). Let **G**_*P*_ = {**g**_*i*_|*θ*_*i*_}_1,…*I*_ denote the probe gait features where *θ*_*i*_ is the view angle of **g**_*i*_, and *I* is the number of the estimated probe view angles. Similarly, let $G_{R}^{m}={\left\{g_{j}^{m}|\theta_{j}\right\}}_{1,\ldots J_{m}}$ denote the gallery gait features of subject *m*, where *J*_*m*_ is the number of the registered gallery angles for subject *m*. The average distance between **G**_*P*_ and $G_{R}^{m}$ can thus be estimated as follows:

$$\begin{array}{c} D\left(G_{P},G_{R}^{m}\right)=\frac{1}{I}\sum_{i=1}^{I}d\left(g_{i},G_{R}^{m}\right) \end{array}$$ (11)

where

$$\begin{array}{c} d\left(g_{i},G_{R}^{m}\right)=\{\begin{array}{cc} & ∥g_{i}-g_{j}^{m}∥\;or\;∥g_{i}-{\hat{g}}_{j|+}^{m}∥,\;\;\;\;\;\;\;\;\;\;\;\;\;\;\;\;\;\;if|\theta_{i}-\theta_{j}|<\epsilon ;\\ & ∥g_{i}-(\alpha_{j_{1}j_{2}|i}^{*}\Phi_{j_{1}}g_{j_{1}}^{m}+\left(1-\alpha_{j_{1}j_{2}|i}^{*}\right)\Phi_{j_{2}}g_{j_{2}}^{m})∥,\\ & \;\;\;\;\;\;\;\;\;\;\;\;\;\;\;\;\;\;\;\;\;\;\;\;\;\;\;\;\;\;\;\;\;\;\;\;\;\;\;\;\;\;\;\;\;\;\;\;\;\;\;\;\;\;\;\;\;\;\;\;otherwise. \end{array} \end{array}$$ (12)

where $\theta_{j_{1}}$ and $\theta_{j_{2}}$ are two registered gallery view angles closest to *θ*_*i*_ for subject *m*, with $\theta_{j_{1}}$<*θ*_*i*_<$\theta_{j_{2}}$, ${\hat{g}}_{j|+}^{m}$ is calculated from the available gallery gait features of subject *m* using Eq (6), and *ϵ* is a threshold of the angle difference so that the gait features from the two angles can be directly compared without the risk of the obvious degradation of recognition accuracy (Note *ϵ* = *Th*_*θ*_ in our experiments).

**Algorithm 2**: The Joint Gait Manifold (JGM) algorithm.

**Input**: the probe gait features **G**_*P*_ = {**g**_*i*_|*θ*_*i*_}_1,…*I*_ from a probe gait sequence,

   the gallery gait features $G_{R}^{m}={\left\{g_{j}^{m}|\theta_{j}\right\}}_{1,\ldots J_{m}}$ of subject *m*,

   the pre-constructed view transformation matrix **P** = [**P**_1_, …, **P**_*N*_]^T^;

**Output**: the distance $D(G_{P},G_{R}^{m})$.

**for** *i* = 1 to *I* **do**

 **if** ∣*θ*_*i*_ − *θ*_*j*_∣< *ϵ* where $\theta_{j}\in {\left\{\theta_{k}\right\}}_{1,\ldots J_{m}}$ **then**

  **if** $\exists g_{j}^{m}$ **then**

$d\left(g_{i},G_{R}^{m}\right)=∥g_{i}-g_{j}^{m}∥$;

  **else**

   Calculate the transformed feature ${\hat{g}}_{j|+}^{m}$ from the available gallery gait features of subject *m* using Eq 6;

$d\left(g_{i},G_{R}^{m}\right)=∥g_{i}-{\hat{g}}_{j|+}^{m}∥$;

  **end**

 **else**

  Let $\theta_{j_{1}}$ and $\theta_{j_{2}}$ denote the two gallery view angles closest to *θ*_*i*_;

  Calculate the mapping matrices $\Phi_{j_{1}}=P_{i}P_{j_{1}}^{+}$ and $\Phi_{j_{2}}=P_{i}P_{j_{2}}^{+}$;

  Find the optimal value $\alpha_{j_{1}j_{2}|i}^{*}$ using Eq 10;

$d\left(g_{i},G_{R}^{m}\right)=∥g_{i}-(\alpha_{j_{1}j_{2}|i}^{*}\Phi_{j_{1}}g_{j_{1}}^{m}+\left(1-\alpha_{j_{1}j_{2}|i}^{*}\right)\Phi_{j_{2}}g_{j_{2}}^{m})∥$;

 **end**

**end**

$D\left(G_{P},G_{R}^{m}\right)=\frac{1}{I}\sum_{i=1}^{I}d\left(g_{i},G_{R}^{m}\right))$;

The computational complexity of this algorithm mainly lies on the offline construction of the view transformation matrix and the online optimization of the joint manifold weight $\alpha_{j_{1}j_{2}|i}^{*}$. Basically, the construction of the view transformation matrix is determined by the Truncated SVD (TSVD), which requires the computational complexity of $O(min\left(M^{2}N,MN^{2}\right))$ at most where *M* and *N* are the numbers of the training subjects and views. For the calculation of $\alpha_{j_{1}j_{2}|i}^{*}$, at most 10 times of distance computations are needed, totally with the computational complexity of $O(N_{g}^{3})$ where *N*_*g*_ is the dimension of each gait feature. Thus the online computational complexity of this algorithm is approximate to $O(IN_{g}^{3})$ for each probe gait sequence. Overall speaking, the JEM algorithm is computationally efficient.

### RankSVM-based gallery data supplementing

In real-world surveillance scenes, it is often difficult to collect and annotate the gait data from all pre-defined views. This is especially true for some specific subject, e.g., criminal suspects. As a result, we may not be able to find the gallery data whose views are close to the probe view. Obviously, a large difference between probe views and gallery views would lead to performance drop [5, 11]. To address this problem, we develop a RankSVM-based algorithm that supplements gait data when gallery features are only available in certain pre-defined views.

In order to approximate the gallery gait feature $g_{i}^{m}$ of subject *m* under a specific view angle *θ*_*i*_, we make use of the gallery gait features ${\left\{g_{i}^{n}\right\}}_{n\in A(m)}$ of the neighboring subjects A(m) (referred to as *view-intrinsic approximation*), as well as other gallery gait features $\left\{g_{j}^{m}\right\}$ of subject *m* that are closest to *θ*_*i*_ (referred to as *subject-intrinsic approximation*). According to the discussion in the previous section, the subject-intrinsic approximation can be obtained by Eq 7. While for view-intrinsic approximation, we need to obtain A(m) in the case of $g_{i}^{m}$ being missed. In [49], a View Feature Recovering Model (VFRM) was proposed to recover the missing data with the average of the gait features of *K*-nearest-neighboring subjects, where the neighborhood was measured using the Geodesic distance. Instead of using *unsupervised* models, this study utilizes *supervised* learning-to-rank methods to learn the neighborhood among subjects, which has been successfully applied in person re-identification [50] and gait recognition [51].

Let $G^{m}=\{g_{u}^{m}\}$ denote the gallery gait features of subject *m* where $g_{i}^{m}$ is missed, and similarly $G^{n}=\{g_{v}^{n}\}$ for subject *n* but $g_{i}^{n}$ exists. Here we assume that when the gait feature of a subject is unavailable under *θ*_*i*_, there exists at least one of other subjects whose gait data under *θ*_*i*_ are available in the gallery dataset. Let $\left\{\theta_{j_{1}},\theta_{j_{2}},\ldots ,\theta_{j_{x}}\right\}$ denote the common view angles between **G**^*m*^ and **G**^*n*^, the objective of the learning-to-rank model is to learn the neighborhood rank between *m* and *n*, denoted by $y^{n,m}=R(|G^{n}-G^{m}\left|\right)$, by utilizing the gait features from these common views, namely,

$$\begin{array}{c} y^{n,m}=f\left(\Delta^{n,m}\right), \end{array}$$ (13)

where $\Delta^{n,m}={\left[|g_{j_{1}}^{n}-g_{j_{1}}^{m}\left|,\ldots ,\right|g_{j_{x}}^{n}-g_{j_{x}}^{m}|\right]}^{T}$ denotes the entry-wise difference matrix between the gait features of *m* and *n* under $\left\{\theta_{j_{1}},\theta_{j_{2}},\ldots ,\theta_{j_{x}}\right\}$, and *f*() is the ranking scoring function. With **Δ**^*n*,*m*^ as the input, the learning-to-rank model outputs a ranking score *y*^*n*,*m*^. If *y*^*n*_1_,*m*^≻*y*^*n*_2_,*m*^, subject *n*_1_ is nearer to subject *m* than subject *n*_2_.

This model can be further decomposed into several sub-problems, each of which corresponds to a gait feature ranking problem. Let $\Delta_{t}^{n,m}$ denote the *t*^*th*^ row of **Δ**^*n*,*m*^, i.e., $\Delta_{t}^{n,m}=\left|g_{t}^{n}-g_{t}^{m}\right|$, the scoring function becomes:

$$\begin{array}{c} f\left(\Delta^{n,m}\right)=\frac{1}{j_{x}}\sum_{t}f\left(\Delta_{t}^{n,m}\right)=\frac{1}{j_{x}}\sum_{t}w^{T}\Delta_{t}^{n,m}, \end{array}$$ (14)

where **w** indicates the importance of the feature distances in measuring the neighborhood and it can be shared across different views. Similar to [50, 51], RankSVM is used to learn the optimal **w** since it is suitable for a large-scale learning problem even with more missing data. Technologically, RankSVM aims to solve the following optimization problem [51]:

$$\begin{array}{c} \begin{array}{c} \frac{1}{2}{∥w^{T}∥}^{2}+C\sum_{p=1}^{\left|P\right|}\xi_{p}\\ \begin{array}{ccc} s.t. & w^{T}\left(\Delta_{p}^{+}-\Delta_{p}^{-}\right)\geq 1-\xi_{p}, & \xi_{p}>0, \end{array} \end{array} \end{array}$$ (15)

where *p* is the index of the preference pairs $\left\{\left(\Delta_{p}^{+},\Delta_{p}^{-}\right)\right\}$, $\Delta_{p}^{+}$ (or $\Delta_{p}^{-}$) is the difference vector between a gait feature **g**_*p*_ and its matching feature $g_{p}^{+}$ (or non-matching feature $g_{p}^{-}$), |*P*| is the total number of the preference pairs used for training, *C* is a positive importance weight on the ranking performance, and *ξ*_*p*_ is the hinge loss used in SVM. Note that the preference pairs can be constructed by treating each gait sequence in the training set as **g**_*p*_ while all the remaining gait sequences in the same view as eithor $g_{p}^{+}$ or $g_{p}^{-}$ depending on its relevance indicator with respect to **g**_*p*_. By going through all pairs, this problem can be efficiently solved with the Newton method.

After obtaining the neighborhood of subject *m*, $g_{i}^{m}$ can be approached by a combination of subject-intrinsic and view-intrinsic approximations as follows:

$$\begin{array}{c} {\tilde{g}}_{i}^{m}\approx (\Phi_{i_{1}}g_{i_{1}}^{m}+\Phi_{i_{2}}g_{i_{2}}^{m})+\frac{\lambda }{K}\sum_{n\in A(m)}g_{i}^{n}, \end{array}$$ (16)

where λ is the weighting factor of the two approximations (λ = 0.1 in our system), and other symbols are defined above. Extensive evaluations validate the effectiveness of this gallery data supplementing algorithm, to be discussed in the following section.

## Experiments

### Experimental setups

#### Datasets

The proposed technique is evaluated and benchmarked with the state-of-the-art over two gait datasets: CASIA gait dataset B [11] and PKU HumanID [12].

The **CASIA gait dataset B** (CASIA-B) contains 124 subjects from 11 view angles (0°, 18°, 36°, 54°, 72°, 90°, 108°, 126°, 144°, 162°and 180°). For each subject under each view, there are 10 walking sequences consisting of 6 normal walking sequences denoted by **NM**, 2 carrying-bag sequences denoted by **BG** and 2 wearing-coat sequences denoted by **CT**. This databset was originally designed to evaluate multi-view gait recognition under view changes. In order to evaluate the recognition performance on a controlled free-view scene, two variants were constructed by: 1) manually removing the training and gallery data corresponding to the probe view and its mirroring view (called the view missing variant); and 2) by randomly removing different proportions of the training and gallery data (called the data missing variant). Since frontal and back views provide little gait information, the gait sequences from 11 views (i.e., from 0°-180°) were used for view estimation, but only sequences from 9 views (i.e., from 18°-162°) for gait recognition.

**Fig 8:**
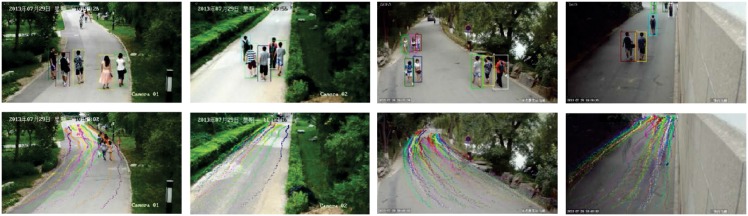
Examples from the PKU dataset: The first row shows sample images with labelled pedestrians in cameras HD01, HD02-1, WMHD-1 and YTX-1, and the second row shows the corresponding pedestrian centroid trajectory.

The **PKU HumanID Dataset (PKU)** is an outdoor dataset captured in a completely uncontrolled environment. It consists of videos of 18 labeled subjects across 11 cameras on a campus where all subjects are masked with bounding box manually. As subjects are walking freely and unpredictably, some subject may not appear in all cameras and it is almost impossible to collect the gait features of each subject across all view angles. Moreover, gait sequences from several cameras (e.g., HD04, HD05 and XDMN) were excluded due to the existence of highly-cluttered background and highly-disordered pedestrian movement. Fig 8 shows several sample images of the labeled subjects and their walking trajectories in this dataset. Table 1 briefly describes all these sequences. In our experiments, sequences of 8 subjects from all cameras were used for training, while the sequences of the remaining 10 subjects were used for testing.

**Table 1:** A brief description of different gait sequences in the PKU dataset.

Camera	Persons	Labeled subject	View
HD01	30	3, 6, 7, 12, 13	back
HD02-1	33	1, 2, 3, 4, 6, 7, 8, 9, 12, 13	back
HD02-2	65	1, 2, 3, 4, 6, 7, 8, 9, 12, 13, 14, 15, 16, 17, 18	front
BWBQ	51	5, 7, 9, 11, 12, 13, 14, 15, 16, 17, 18	front
DCM	87	1, 7, 11, 12, 13, 14, 15, 16, 17, 18	front
WMHD-1	139	1, 2, 3, 4, 6, 7, 8, 9, 11, 12, 13, 14, 15, 16, 17, 18	front
WMHD-2	66	1, 2, 3, 4, 6, 7, 8, 9, 11, 12, 13, 14, 15, 16, 17, 18	back
YTX-1	150	1, 2, 3, 4, 6, 7, 8, 9, 11, 12, 13, 14, 15, 16, 17, 18	back
YTX-2	73	1, 2, 3, 4, 6, 7, 8, 9, 11, 12, 13, 14, 15, 16, 17, 18	front

#### Setups

Two sets of experiments were designed as listed:

1. The first set was designed to evaluate the recognition performance in controlled free-view scenes. It was performed on the CASIA-B dataset.
2. The second set was designed to evaluate the recognition performance in uncontrolled free-view scenes. It was performed on the PKU dataset.

All experiments were performed on a PC sever with 2.0GHz CPU and 2G RAM.

### Free-view gait recognition in controlled scenes

This set of experiments evaluate the free-view recognition of our VAM in controlled scenes. These experiments were performed on the two variants of CASIA-B, where 24 subjects were used for training and the rest 100 subjects were used for testing.

**Fig 9:**
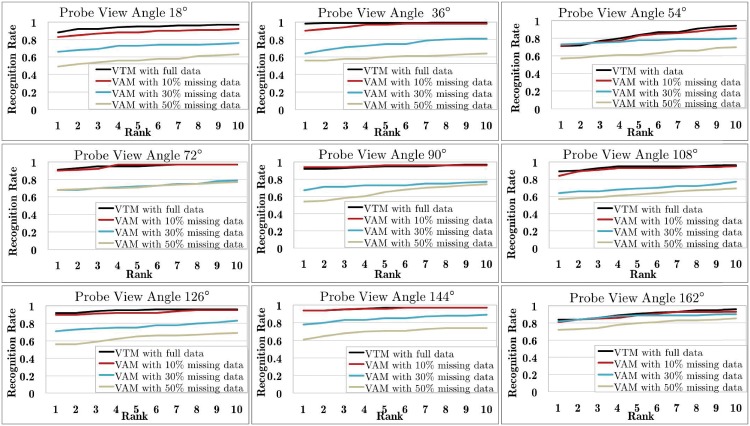
Results of free-view gait recognition in the *data missing* variant of CASIA-B, where different proportions of the training and gallery data were randomly abandoned.

The first experiment is conducted over the data missing variant of CASIA-B, where different proportions of the training and gallery data (here 10%, 30% and 50%) are randomly abandoned. The objective is to evaluate the VAM robustness by simulating a common setting of free-view gait recognition. The improved VTM [9] with full data was used for comparison. Fig 9 shows experimental results. We can see that VAM with 10% missing data gives similar performance as the VTM trained with full data. When the proportion of missing data reaches 30% and 50%, the VAM recognition rate is even higher than 0.6 in most cases. This shows that VAM has better robustness when only partial training and gallery data are available.

**Fig 10:**
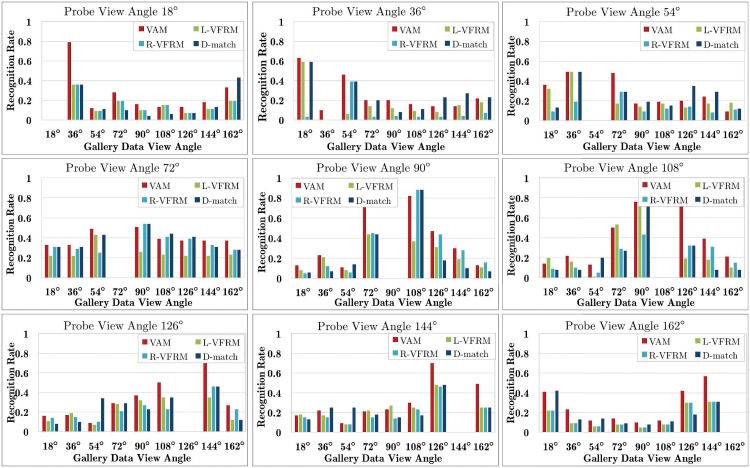
Results of free-view gait recognition where only one view (i.e., marked in the x-coordinate) is kept while the probe view and 50% data of the other views are missing in the training and gallery sets of CASIA-B.

The second experiment is conducted over a more challenging case by combining the view missing and data missing variants of CASIA-B, i.e., only one view is kept while the probe view and 50% data of the other views are missing in the training and gallery sets. Since only data in one view is completely available in the training set, two variants of VFRM [49] (i.e., L-VFRM and R-VFRM which are similar to L-VTM and R-VTM except that they utilize the GKNN-based algorithm to recover the incomplete training data and then generate the VTM matrix) and D-match [11] (direct matching across two views using gait features) are used for comparison. Experimental results are shown in Fig 10, where the x-coordinate denotes the current gallery view angle whose data is kept in the training set. We can see that the gait recognition degrades heavily compared with the results in Fig 9. This is reasonable as the available training data are much less and the task actually becomes a cross-view recognition problem. Even in this case, the proposed VAM still outperforms the other three baseline methods clearly.

### Free-view gait recognition in uncontrolled scenes

The last experiment is to evaluate the free-view gait recognition of the VAM on the PKU dataset. In this real-world free-view dataset, subjects walk freely while each subject may not appear at all cameras. Thus all components in the VAM should work together to complete the recognition process. To the best of our knowledge, this is the first work for free-view gait recognition in uncontrolled scenes. We modified several existing gait recognition methods by adding certain modules (e.g., view angle estimation) and use them as the reference methods. They include D-match [11], CCA [10], PrRankSVM [51], and VTM [9]. Among them, CCA and PrRankSVM are two state-of-the-art cross-view gait recognition methods, while VTM is a modified version of the original VTM [9] by directly selecting the nearest gallery view to the probe data for view transformation as approximation [20].

**Fig 11:**
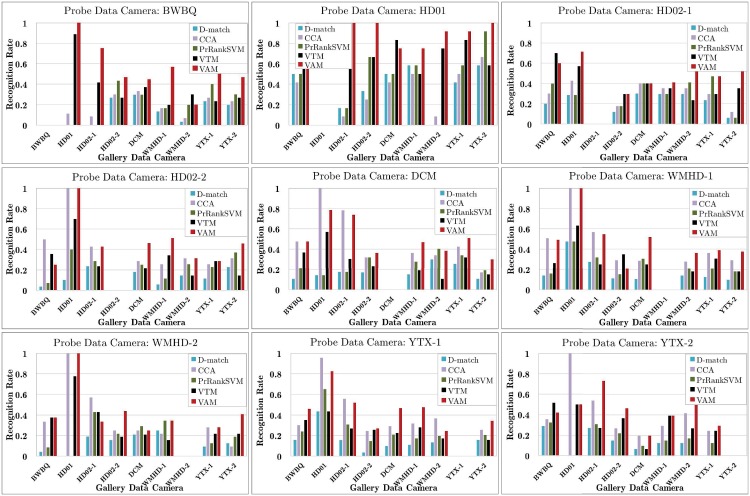
Results of free-view gait recognition on the PKU dataset.

Even in the view field of the same camera, a person may walk freely, and his/her walking directions may change randomly. Thus in each trial, we selected the gait sequences from one camera as the probe data and the sequences from other cameras as the gallery data. Fig 11 shows experimental results. We can see that VAM achieves the highest recognition rate on average, and outperforms other compared methods remarkably. This validates the VAM’s effectiveness for free-view gait recognition.

Due to the lack of sufficient training data, some reference method may perform worse than the baseline (i.e., D-match). Among them, CCA exhibits relatively good recognition performance in most cases, except when BWBQ and HD01 were treated as the probe data camera. It should be noted that all compared methods including our VAM perform worse on this dataset as compared with CASIA-B. Additionally, the recognition rates are overall very low and unsuitable for applications in real-world free-view scenes. There is still a long way towards usable free-view gait recognition.

### Discussion

The performance of the VAM depends heavily on our designed view angle estimation, joint gait manifold and gallery data supplementing. We evaluate the three designs one by one, where the view estimation is evaluated over both CASIA-B and PKU datasets and the other two are evaluated on the CASIA-B only.

#### View angle estimation

The proposed WTF was compared with GP-based and SVM-based methods [10]. The gait sequences in **NM**, **BG** and **CT** of CASIA-B were divided into two equally-size subsets, one for training and the other for testing. Table 2 shows experimental results, where the proposed WTF obtains clear better results than SVM-based and GP-based classifiers. In particular, it achieves satisfactory results under all co-variate conditions and across all views, and even works well for the cases of frontal view (0°and 18°) and back view (162°and 180°). Note the SVM-based classifier gets poor results for some views (e.g., 54°, 108°and 126°) due to its limited robustness.

**Table 2:** View estimation results (%) for each view on CASIA-B.

Case	Method	0°	18°	36°	54°	72°	90°	108°	126°	144°	162°	180°	AVG
NM	WTF	99.0	98.6	91.0	96.3	87.0	89.0	91.0	98.9	82.3	100.0	100.0	**93.92**
	GP	-	-	84.0	91.2	85.3	74.0	86.0	91.2	93.5	-	-	**86.46**
	SVM	-	-	94.9	40.5	85.4	64.3	24.0	43.6	98.0	-	-	**64.39**
BG	WTF	100.0	95.0	86.0	95.9	91.0	90.0	90.0	89.0	82.3	96.9	100.0	**92.37**
	GP	-	-	83.4	88.7	84.9	68.6	83.0	92.7	93.5	-	-	**84.97**
	SVM	-	-	96.1	41.8	79.3	62.6	28.1	50.6	97.9	-	-	**65.20**
CT	WTF	100.0	96.0	88.0	90.1	87.1	85.7	87.6	98.0	87.8	99.0	98.0	**92.48**
	GP	-	-	84.0	91.2	85.3	74.0	86.0	91.2	93.5	-	-	**86.46**
	SVM	-	-	93.7	50.0	81.0	61.2	22.5	41.5	96.6	-	-	**63.79**

Table 3 shows the view angle estimation results on the PKU dataset, where the WTF outperforms the SVM-based and GP-based methods as well. Due to the very low quality of the pedestrians’ silhouettes, both SVM-based and GP-based methods do not perform well and they also fail to recognize view angles when heavy occlusions exist (e.g. Cameras DCM and WMHD). As a comparison, the WTF is much more robust even with heavy occlusions as far as the subjects’ walking trajectories are identified explicitly.

**Table 3:** View estimation results (%) for each camera on the PKU database.

Method	HD01	HD02-1&2	BWBQ	DCM	WMHD-1&2	YTX-1&2	AVG
WTF	100.0	81.8	81.3	96.8	88.9	81.5	**88.38**
GP	50.0	45.5	62.5	29.0	37.0	51.8	**45.97**
SVM	50.0	63.6	56.3	12.9	14.8	66.7	**44.05**

To evaluate how the view angle estimation will affect the gait recognition performance, we test the VAM by using the *ground-truth* or *estimated* view angles of the probe data. Experiments show that the gain in the Rank-1 recognition is less than 1% on the CASIA-B and 2% on the PKU on average. This validates that better view angle estimation does help for better gait recognition though the gain is not significant. There are two possible reasons. First, our view angle estimation is based on the analysis of pedestrian’s walking trajectory that can guarantee differences between the estimated angles. If wrongly estimated, the corresponding ground-truth (or its mirroring view angle in CASIA-B) should be quite small. Second, if the wrongly-estimated view angle is close to the corresponding ground-truth or its mirroring view angle, the VAM can learn an optimal manifold for gait similarity evaluation.

#### Joint gait manifold

The joint gait manifold (JGM) evaluate the gait similarity between the probe and relevant gallery data by using the optimal joint manifold that is constructed from two closest reference view angles $\theta_{j_{1}}$ and $\theta_{j_{2}}$ (here $\theta_{j_{1}}<\theta_{i}<\theta_{j_{2}}$) to the probe view angle *θ*_*i*_. Similar to the discrete view transformation [20], two baseline methods were benchmarked: the VTM [9, 46] that transforms the gallery gait features to $\theta_{j_{1}}$ (denoted as L-VTM), and the VTM that transforms the gait features to $\theta_{j_{2}}$ (denoted as R-VTM). To test whether more than two reference views are better for gait recognition, we also extended the JGM with four or eight reference views as denoted by JGM_4 and JGM_8, respectively. For JGM_4, the gallery gait features from view angles *θ*_*i*_ − 36, *θ*_*i*_ − 18, *θ*_*i*_ + 18 and *θ*_*i*_ + 36 are used. All evaluations are performed over the view missing variant of CASIA-B.

**Fig 12:**
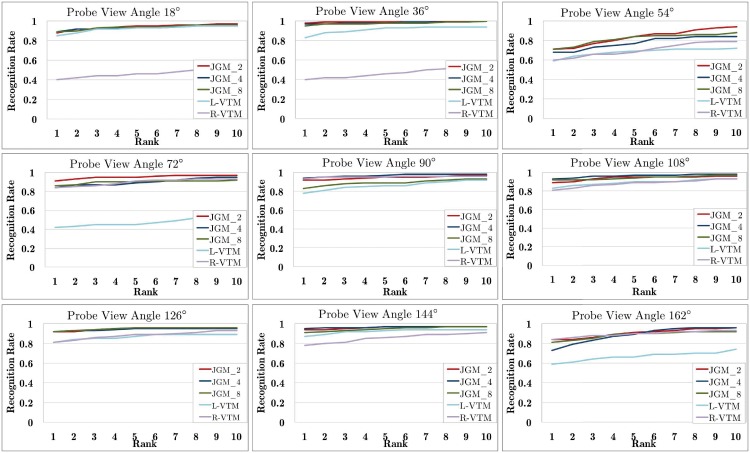
Gait recognition results using JGM and VTM on the CASIA-B, where L-VTM and R-VTM are two implementation versions of VTM [9, 46] and JGM_*n* denotes the JGM with *n* reference views (*n* = 2, 4, 8).

Experimental results are shown in Fig 12. As Fig 10 shows, all JGM versions outperform the two VTM variants remarkably. This validates the effectiveness of the proposed JGM, and also shows that simply choosing the closest view to the probe data for view transformation [20] is not optimal. Moreover, JGM_2, JGM_4 and JGM_8 produce very similar recognition, meaning that including more reference views won’t improve JGM much. It is thus reasonable to select two reference views for the JGM. We also observe that the recognition rate of the JGM at rank 1 is pretty good and even comparable to that at top 10 in most cases.

In terms of efficiency, we have shown that the JGM algorithm only include some operation over the VTM for online optimizing the joint manifold weight. It incurs the additional computational complexity of $O(IN_{g}^{3})$ for each probe gait sequence that consists of *I* gait features extracted from different view angles. Considering that the magnitude of *N*_*g*_ is not so big in practical applications, this additional computation is fair and manageable. Our experiments also show that it takes additional computational cost of 500ms to 2s for different probe gait sequences.

#### Gallery data supplementing

**Fig 13:**
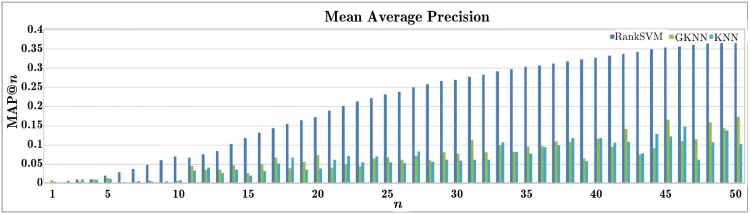
mAP in the gallery data supplementing experiment where data from a random view angle and its mirroring view are discarded for each subject.

The objective of this experiment is to evaluate the effectiveness of our RankSVM-based gallery data supplementing. Two baseline methods, GKNN and KNN [49], were used for comparison. The first experiment was conducted over the view missing variant of CASIA-B, where the discarded data is used as the probe in each trial while the supplemented data as the gallery. For a set of queries, Mean Average Precision (mAP) is defined as the mean of the average precision scores [52]. Fig 13 shows the *mAP*@*n* results for *n* ≤ 50. We can see that the results of RankSVM are much better than those of GKNN and KNN which means better recognition could be expected when using RankSVM to supplement the missing data.

The second experiment is performed on the data missing variant of CASIA-B, where different proportions of data were discarded randomly (here 10%, 30%, and 50%). In this case, the recovering error rate is used for evaluation:

$$\begin{array}{c} r=\frac{\sum_{v=1}^{V}\sum_{m=1}^{M}∥{\tilde{g}}_{v}^{m}-g_{v}^{m}∥}{\sum_{v=1}^{V}\sum_{m=1}^{M}∥g_{v}^{m}∥} \end{array}$$ (17)

where ${\tilde{g}}_{v}^{m}$ and $g_{v}^{m}$ are the supplemented and original gait features for subject *m* under the view angle *θ*_*v*_. A smaller *r* means higher similarity between the supplemented and original data. As shown in Table 4, RankSVM outperforms both GKNN and KNN remarkably, especially for a large missing proportion. RankSVM thus shows higher robustness in dealing with the gallery data supplementing problem.

**Table 4:** Recovering error rates when different proportions of gallery data are missing.

Method	Percent of missing data		
	10%	30%	50%
RankSVM	0.026	0.029	0.039
GKNN	0.026	0.074	0.098
KNN	0.025	0.077	0.111

## Conclusion

This study identifies *free-view* gait recognition as a new type of gait recognition challenge in the real-world scenes, where gait sequences are captured from uncontrolled scenes and the probe view angles are unknown, dynamically changing, or without pre-defined views in the gallery dataset. We propose a novel view-adaptive mapping (VAM) approach to address these challenges. Specifically, VAM designs walking trajectory fitting to estimate the view angles of a gait sequence, joint gait manifold to approximate the unknown probe manifold, and RankSVM-based algorithm to supplement the gallery data for subjects whose gallery features are partially available. Experiments on indoor and outdoor datasets demonstrate the superior performance of the proposed VAM under the free-view gait recognition setting.

Moving forwards, we will further improve the VAM and verify its effectiveness in larger and more challenging datasets. One promising direction is to introduce deep features into the gait recognition framework to improve the recognition accuracy and robustness, targeting applications in real-world gait recognition in the near future.
